# A Novel Deep Transfer Learning-Based Approach for Automated Pes Planus Diagnosis Using X-ray Image

**DOI:** 10.3390/diagnostics13091662

**Published:** 2023-05-08

**Authors:** Yeliz Gül, Süleyman Yaman, Derya Avcı, Atilla Hikmet Çilengir, Mehtap Balaban, Hasan Güler

**Affiliations:** 1 Department of Radiology, Elazig Fethi Sekin City Hospital, 23280 Elazig, Turkey; 2 Biomedical Department, Vocational School of Technical Sciences, Firat University, 23119 Elazig, Turkey; suleyman.yaman@firat.edu.tr; 3 Department of Software Engineering, Technology Faculty, Firat University, 23119 Elazig, Turkey; 4 Department of Radiology, Faculty of Medicine, Izmir Democracy University, 35140 Izmir, Turkey; 5 Department of Radiology, Faculty of Medicine, Ankara Yildirim Beyazit University, 06010 Ankara, Turkey; 6 Electrical-Electronics Engineering Department, Engineering Faculty, Firat University, 23119 Elazig, Turkey

**Keywords:** pes planus, deep learning, transfer learning, iterative ReliefF, pyramidal feature extraction, X-ray image classification

## Abstract

Pes planus, colloquially known as flatfoot, is a deformity defined as the collapse, flattening or loss of the medial longitudinal arch of the foot. The first standard radiographic examination for diagnosing pes planus involves lateral and dorsoplantar weight-bearing radiographs. Recently, many artificial intelligence-based computer-aided diagnosis (CAD) systems and models have been developed for the detection of various diseases from radiological images. However, to the best of our knowledge, no model and system has been proposed in the literature for automated pes planus diagnosis using X-ray images. This study presents a novel deep learning-based model for automated pes planus diagnosis using X-ray images, a first in the literature. To perform this study, a new pes planus dataset consisting of weight-bearing X-ray images was collected and labeled by specialist radiologists. In the preprocessing stage, the number of X-ray images was augmented and then divided into 4 and 16 patches, respectively in a pyramidal fashion. Thus, a total of 21 images are obtained for each image, including 20 patches and one original image. These 21 images were then fed to the pre-trained MobileNetV2 and 21,000 features were extracted from the Logits layer. Among the extracted deep features, the most important 1312 features were selected using the proposed iterative ReliefF algorithm, and then classified with support vector machine (SVM). The proposed deep learning-based framework achieved 95.14% accuracy using 10-fold cross validation. The results demonstrate that our transfer learning-based model can be used as an auxiliary tool for diagnosing pes planus in clinical practice.

## 1. Introduction

Pes planus is a multidirectional deformity that occurs with the collapse of the medial longitudinal arch of the foot, valgus deformity of the heel, and forefoot abduction [1]. Its prevalence is between 20–37% in studies among healthy volunteers [2]. Pes planus can be developmental or acquired. Developmental pes planus is considered physiological up to 3 years of age. It may develop in childhood due to immaturity, laxity syndromes, or tarsal coalition. Acquired pes planus is seen after skeletal maturation and develops secondary to reasons such as posterior tibial tendon degeneration, neuromuscular disease, trauma, and arthritis [3]. Pes planus clinic can be asymptomatic, as well as cause pain in the medial arch of the ankle and foot, deterioration in gait, postural disorders in individuals, and various problems in the musculoskeletal system [1,4]. Additionally, the condition can significantly decrease the shock absorption capacity of the feet, increasing the risk of developing various health complications, including toe deformity, and ankle arthritis [5]. Therefore, the correct diagnosis of pes planus is very important. Diagnosis of pes planus involves a physical examination and radiological evaluation. An example of a physical examination is gait observation, where the individual is evaluated both barefoot and wearing shoes, enabling the physician to analyze various signals and characteristics. Medical imaging modalities such as radiographs, computed tomography (CT), magnetic resonance imaging (MRI), and bone scanning have an important place in determining the severity of pes planus, identifying the underlying cause and lesions that can be surgically corrected, and in the diagnosis and treatment decision [6]. Specifically, weight-bearing lateral and dorsoplantar radiographs are typically the first-choice radiographic examinations [7]. Additionally, cross-sectional imaging can provide more detailed anatomical information in patients with advanced pes planus. Lateral radiographs offer better visualization of the medial longitudinal arch and talonavicular joint. In lateral projections, the talus–first metatarsal angle (Meary’s angle), calcaneal inclination angle, lateral talocalcaneal angle, and navicular index can be evaluated [3,4]. The calcaneal inclination angle is the angle between the line drawn from the most plantar point of the calcaneus to the inferior border of the calcaneocuboid joint and the horizontal plane line. It has an important place in the evaluation of the medial longitudinal arch of the foot [3].

Today, artificial intelligence (AI) and especially deep learning (DL) techniques are successfully applied in a wide range of fields, including medicine [8], industry [9], defense industry [10], and the automotive sector [11]. DL, and in particular its flagship, Convolutional Neural Networks (CNNs), have shown tremendous success in classifying and segmenting radiological images in the medical field, outperforming traditional image processing methods [12]. As a result, its adoption has grown in various medical domains, such as radiology [13,14,15], dermatology [16,17], neurology [18,19,20,21], and cardiology [22,23]. AI-based computer-aided diagnosis (CAD) systems reduce the workload of the physician and provide valuable support in the decision-making process. Additionally, these systems minimize the risk of misinterpretation, thus providing more accurate and effective treatment [24,25]. In the broader context of X-ray image analysis, several closely relevant works have been reported in the literature, focusing on different medical conditions. For instance, Rajpurkar et al. [26] proposed a deep learning-based model called CheXNet for pneumonia detection from X-ray images. The authors reported that their model exceeded the performance of an average radiologist in detecting pneumonia. Similarly, Zhou et al. [27], developed two contrastive attention models which demonstrated remarkable success in the automated diagnosis of thoracic diseases from X-ray images. Another notable example is the work by Zhou et al. [28], who proposed two methods, many-to-one distribution learning (MODL) and K- nearest neighbor smoothing (KNNS) for thoracic disease identification, achieving improved performance in disease recognition. In addition, Joshi et al. [29] proposed a robust deep learning-based system for the detection of COVID-19 using chest X-ray images. This system utilized the DarkNet-53 backbone network and transfer learning and achieved impressive average test accuracies of 97.11% for multi-class classification and 99.81% for binary classification. Furthermore, a recent study [30] developed a new necrosis rate detection method for bone tumors using time series X-ray images, which could overcome the limitations of few-shot samples. The proposed method utilized a Generative Adversarial Network with Long Short-term Memory and a 3D-Convolutional Neural Network classification model, providing an efficient alternative to biopsy-based techniques. Another interesting application of deep learning in X-ray image analysis is presented by a study [31] that developed a fully automated deep learning pipeline using digital radiographs to detect the proximal femur region for accurate automated sex estimation. The model, based on convolutional neural networks, achieved an accuracy similar to that of current state-of-the-art mathematical functions using manually extracted features for the Chinese Han population samples, proving to be a reliable choice for human sex estimation. These works employ advanced deep learning techniques to effectively recognize and diagnose various medical conditions, demonstrating the potential of such methods for medical image analysis. Although these studies do not directly focus on pes planus diagnosis, they provide valuable insights into the development of efficient deep learning models for X-ray image analysis. The methodologies and techniques employed in these works can potentially be adapted and fine-tuned to develop a high-accuracy CAD application for automated pes planus diagnosis from X-ray images.

Diagnosis of pes planus presents several challenges that need to be addressed to ensure accurate and timely identification of the condition. Traditional methods of diagnosing pes planus often rely on subjective assessments, such as clinical examination, radiographic imaging, and manual evaluation of foot posture [32,33]. These approaches are subject to inter-observer variability and can be influenced by factors such as the clinician’s experience, training, and personal biases [5,34,35]. For instance, Yildiz et al. [35] conducted a clinical study to examine inter-observer reliability in the diagnosis of flatfoot. In the study, two groups of four medical specialists, two radiologists and two orthopedists, were formed. The formed two groups evaluated weight-bearing X-ray images for the diagnosis of pes planus using eight parameters. According to the results of the evaluation, there were great differences between the specialists, especially in the determination of the longitudinal arch angle. Furthermore, such assessments can be time-consuming, costly, and may require multiple visits to healthcare facilities, posing significant challenges to both patients and healthcare providers. Diagnosis of pes planus from X-ray images is a laborious and time-consuming process for specialists [7,36,37,38,39]. Therefore, it is essential to explore innovative diagnostic methods that can address these limitations, and ultimately improve patient outcomes. 

Recent advancements in machine learning and deep learning algorithms offer promising solutions for the automated diagnosis of pes planus, enabling more accurate and objective assessments. Various studies have been carried out in the literature to assist the specialist in the decision-making process, to reduce the workload and to diagnose correctly. A few, albeit limited, CAD systems have been developed in diagnosing pes planus to overcome these challenges and assist specialist in the decision-making process. Chen et al. [40] proposed an artificial neural network-based model called WASDNN-DP for the detection of pes planus. They collected dynamic data from children aged 7–15 using smart insoles. The collected data were analyzed in the FreeStep software and 82 hand-crafted features such as momentum and pressure were extracted. Extracted features were classified by WASDNN-DP as either flat foot or not. The hold out validation technique was used in the training of the neural network. Test accuracy of 84.62% and 82.61% was obtained for the right and left feet, respectively. In a similar study, Li et al. [6] introduced a new neural network-based method called MWASDNN, which classifies foot data as pes planus or normal. They collected foot data using smart insoles, and subsequently extracted 82 features through the FreeStep software. These extracted features were fed into MWASDNN for classification. The proposed method achieved an accuracy of 84.31 for the left foot and 85.29% for the right foot, respectively. Ryu et al. [41] examined the accuracy and efficiency of an automated approach for detecting flatfoot landmarks using a state-of-the-art cascade CNN algorithm named Flatfoot Landmarks AnnoTating Network (FlatNet). They collected a total of 1200 consecutive weight-bearing lateral radiographs of the foot, including 1050 radiographs for training and tuning purposes and the 150 radiographs for testing. An expert orthopedic surgeon manually labeled twenty-five anatomical landmarks as ground truths. The results indicated that the newly developed FlatNet algorithm demonstrated superior accuracy and reliability in comparison to human observers. Additionally, the accuracy and reliability of the human observers generally increased under the guidance of FlatNet. Mei et al. [42] introduced a framework for classifying foot types (normal, cavus, and planus) using sensor-equipped insoles and one-dimensional convolutional neural networks (1D-CNN). They developed an insole prototype that incorporated nine force sensors and an inertial sensor to gather both kinematic and kinetic information. A total of 80 subjects participated in the study, and a podiatrist labeled each foot type. The 1D-CNN was employed to classify the collected signals into normal, cavus, and planus classes, and the highest accuracy of 99.26% was achieved using the combination of angular velocity and force sensing. Chae et al. [5] presented a deep learning-based ensemble model that uses image pressure and numerical data to classify foot deformations into concave, normal, and flat feet. The model utilized a fine-tuned VGG-16 architecture for image data and k-NN models for numerical data, which proved to be the most effective in their experimental studies. The predicted values generated by these models were then input into a shallow neural network. The study conducted experiments on data collected from 96 subjects, and the proposed model achieved an F1-score of 92.55%. Kim [43] proposed a transfer learning-based model for flat foot classification. The authors employed a pre-trained VGG16 model and data augmentation techniques on a dataset of 176 images, consisting of 88 flat feet and 88 normal feet. The proposed model achieved a test accuracy of 84.9%. Eksen et al. [44] developed a novel mobile pre-diagnosis system for pes planus and pes cavus, utilizing image processing and deep neural networks in a mobile phone app. The system employs conventional deformity identification methods from the literature. A prototype was tested on 34 participants, and the results were compared to an orthopedic specialist’s findings. The prototype achieved a 91.80% match, indicating its potential for remote identification and classification of foot deformities such as pes cavus and pes planus. Despite some limitations related to image quality and a small number of participants, the prototype showed promise for future development and improvement. Koo et al. [38] conducted a study to determine if a deep learning-based algorithm could improve the reproducibility and diagnostic accuracy of radiographic measurements for pes planus. They used 300 lateral radiographs for algorithm development and 95 for validation. A deep learning algorithm called SegNet was employed to segment the outlines of the bones and identify reference points for angle measurement using a deep learning method. Meary angle (MA) and calcaneal pitch (CP) were measured, and the results showed improved inter-observer agreement when using the algorithm compared to measurements taken without it. The segmentation performance, measured by the dice similarity coefficient, was 96% for the talus, 93% for the first metatarsus, and 98% for the calcaneus. Overall, the study demonstrated that the deep learning algorithm could enhance reproducibility and potentially increase diagnostic accuracy for pes planus measurements. Ryu et al. [24] developed a fully automatic semantic segmentation model based on U-Net to evaluate the relationship angles with flatfoot. In the developed model, 300 consecutive weight-bearing lateral radiographs that they collected were used. For the training of the U-net model, radiographs were manually segmented using ITK-SNAP and labeled by an expert orthopedist. In the labeling process, tarsal and metatarsal bones were used. The authors used the Dice similarity coefficient (DSC) and Hausdorff distance (HD) metrics to evaluate the segmentation results. The best DSC and HD were calculated as 0.964 and 1.292 mm, respectively. Han et al. [45] proposed a deep model called resident network-based conditional generative adversarial nets (RNcGAN) to classify the foot type for the industry. The authors obtained plantar pressure images using the foot scanner system. From the acquired images, the pixel-level state matrix is given as an input to the proposed model. Thus, plantar pressure images were classified into nine foot types, including pes planus. The proposed RNcGAN-based deep model achieved 95.17% accuracy.

In the existing literature, no artificial intelligence-based studies have been found for automated pes planus diagnosis using X-ray images. Therefore, this study aims to develop a high-accuracy CAD application for automated pes planus diagnosis from X-ray images. To achieve this, we propose a novel, lightweight, deep transfer learning-based model. The proposed model adopts the transfer learning strategy, as the collected dataset is not large enough. Initially, eight well-known pre-trained CNN models were utilized as feature extractors. Subsequently, the features extracted from each deep model were evaluated using conventional classifiers. Based on the evaluation results, the best deep feature extractor (MobileNetV2) and classifier (SVM with a cubic kernel) were selected. To improve the model’s accuracy and reduce computational load, the deep features extracted from MobileNetV2 were input into the proposed Iterative ReliefF feature selection algorithm. Finally, the most important features selected were classified into pes planus or normal using the SVM classifier.

### 1.1. Research Gaps

Our comprehensive literature review helped us identify several key research gaps related to automated pes planus detection. The most important of these are presented below.

- To the best of our knowledge, no artificial intelligence-based model has been proposed for automated pes planus detection using X-ray images, which is the standard imaging method for diagnosing pes planus [37].
- There is no publicly available dataset consisting of weight-bearing X-ray images.
- Limited studies have been conducted for automatic pes planus detection using images such as smart insoles [40] and plantar pressure [45], without using X-ray images. However, both the accuracy obtained is low and the datasets used are small.

### 1.2. Main Contributions and Limitations

The main contributions and limitations of this study are presented below.

- A new pes planus dataset was collected, and labeled by the specialist radiologists.
- A novel, lightweight, and high-accuracy hybrid model based on deep learning is proposed for automated pes planus detection using X-ray images.
- With the proposed iterative ReliefF, the computational load of the model has been reduced, thus a lightweight, fast, and robust model has been developed.
- The performance of conventional classifiers, pre-trained deep models and transfer learning approach in detecting pes planus was investigated.
- The accuracy of the model has been increased with the pyramidal feature extraction technique.
- The proposed pyramidal-MobileNetV2-SVM-based hybrid model achieved 95.14% accuracy.
- Since the collected pes planus dataset was not large enough, training from scratch could not be performed due to the overfitting problem.

The rest of the paper is organized as follows: the collected dataset is explained in Section 2. In Section 3, the proposed model and the methods used are introduced. Experimental studies, results, and evaluation criteria are presented in Section 4. Finally, concluding remarks are given in Section 5.

## 2. Dataset

**Figure 1:**
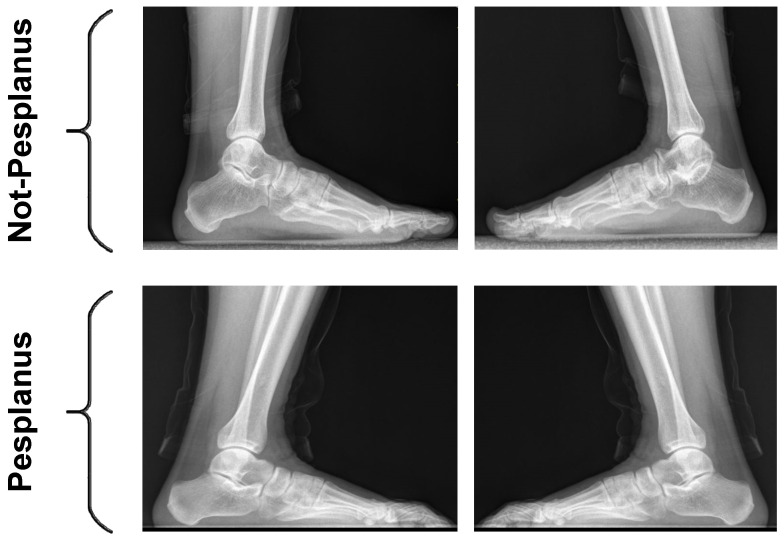
Randomly selected X-ray images from the labeled pes planus dataset.

The weight-bearing lateral X-ray image of the foot provides functional information for diagnosing pes planus [35,41] and it is the gold standard method due to its short acquisition time, cost-effective, and low radiation dose [37]. Therefore, in this study, the weight-bearing X-ray images were used for automated pes planus detection. We retrospectively analyzed weight-bearing X-ray images of patients admitted to the Radiology Department of Elazığ Fethi Sekin City Hospital for routine pre-military health screening or suspected flatfoot and collected a new pes planus dataset consisting of X-ray images of 439 patients aged 14–47 years. These images are stored as JPG images. The collected dataset is available https://www.kaggle.com/datasets/suleyman32/pesplanus-two-class-dataset (accessed on 19 April 2023). Patients with a known neurological disorder, acute orthopedic trauma, or previous orthopedic lower extremity surgery were not included in this study. Images were collected after approval from the ethics committee of Fırat University, Turkey. All images were acquired with Philips, dual detector digital X-ray (65 kV, 6.3 mAs). In the collected pes planus dataset, X-ray images of 18 patients with low quality and resolution were discarded. 842 X-ray images of the remaining 421 patients were labeled by two specialist radiologists by measuring calcaneal inclination angle [3]. A third specialist radiologist performed the post-labeling by re-examining the conflicting examples. In the labeling process, patients with a calcaneal angle below 18 degrees were assigned to the pes planus class, and patients 18 degrees and above were assigned to the normal class. At the end of the labeling process, 440 of the 842 X-ray images were assigned to the normal class and 402 to the pes planus class. Demographics of participants and dataset characteristics is presented in Table 1. Some randomly selected X-ray images from the pes planus and normal classes of the labeled pes planus dataset are shown in Figure 1.

## 3. Methodology

### 3.1. Proposed Method

**Figure 2:**
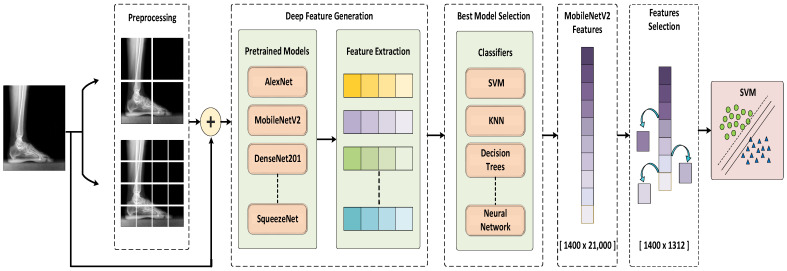
The framework of the proposed method.

This study proposes a novel deep learning-based method for automated pes planus detection from X-ray images. The framework of the proposed method is depicted in Figure 2. The proposed method consists of three stages, namely preprocessing, deep feature generation and the best model selection, iterative feature selection and classification. In the preprocessing step, the number of X-ray images in the dataset is augmented and the images are resized to 512 × 512 × 3. Then, the resized images are divided into patches in a pyramidal fashion before being fed into the CNN models. The process of dividing into patches was carried out for each image in the collected dataset. As a result, a total of 21 images are obtained for each case, one original X-ray image and 20 patches. In the deep feature generation stage, 21,000 deep features are generated for 21 images obtained using pre-trained CNN models. In the next stage, the generated features are fed into conventional machine learning classifier. According to the classification results, the best CNN model (MobileNetV2) and classifier (cubic-SVM) are selected to build a model with higher accuracy. In the feature selection phase, 1312 most important features are selected from the features generated by the MobileNetV2 model using the proposed iterative ReliefF algorithm. Finally, the selected 1312 deep features are classified by the SVM with cubic kernel.

### 3.2. Preprocessing

**Figure 3:**
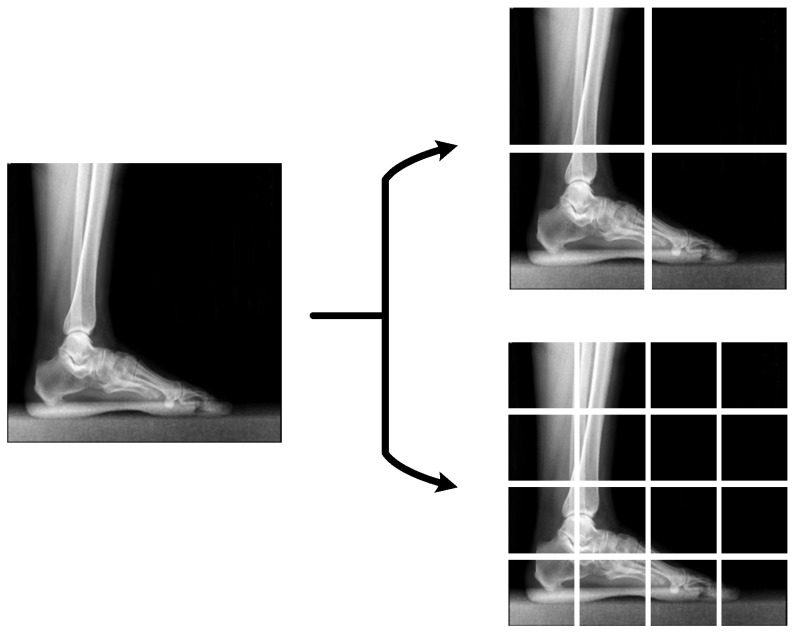
The process of images dividing into patches in a pyramidal fashion.

In the training of machine learning and especially deep learning models, a large dataset is needed to avoid overfitting and to increase the generalization ability of the model [46,47]. However, there is a limited number of labeled datasets in the field of medical imaging. To solve this problem, data augmentation methods aiming to reproduce the data synthetically are used [48,49]. Therefore, in order to avoid the above-mentioned problems, in the preprocessing stage, original X-ray images were synthetically augmented by rotation, scaling and mirroring methods from geometric transformation-based data augmentation techniques [50]. At the end of the data augmentation process, the number of images was augmented from 842 to 1400 and the number of images in each class was balanced to be 700 by 700. In addition, in this study, feature generation is performed in a pyramidal fashion. For this purpose, the X-ray images in the augmented dataset were first resized to 512 × 512 × 3. After that, the resized images were divided into 256 × 256 × 3 and 128 × 128 × 3 sized patches without overlapping as shown in Figure 3. Thus, 4 and 16 patches were obtained for each original X-ray image with dimensions 256 × 256 × 3 and 128 × 128 × 3 sizes, respectively. As a result, a total of 21 images were obtained, including one original X-ray image and 20 patches.

### 3.3. Deep Feature Generation and the Best Model Selection

In this study, a transfer learning strategy was adopted instead of designing a CNN model and training it from scratch, since the size of our pes planus dataset was not large enough. The basic blocks of CNN architecture and transfer learning strategy are explained below.

#### 3.3.1. CNN Architecture

**Figure 4:**
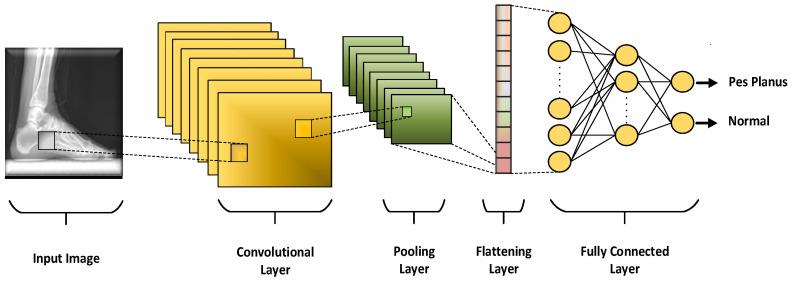
The main blocks of CNN architecture.

CNNs, the flagship of deep learning, are a type of multi-layer perceptron (MLP) inspired by the visual region of animals. CNNs basically consist of four main blocks as convolution layer, pooling layer, flattening layer, and fully connected layer as shown in the Figure 4 [51,52,53]. In the convolution layer, distinctive local features such as edge, color, texture, and gradient orientation of the input image are extracted by using many learnable filters called convolution filters. In the pooling layer, the number of features extracted from the convolution layer is reduced by downsampling by using techniques such as maximum pooling and average pooling. Thus, the computational load of the network is reduced by reducing the size of the feature maps. In the flattening layer, the extracted feature maps are converted to 1-dimensional vector. Finally, in the fully connected layer, features are classified into class labels with classical artificial neural networks.

#### 3.3.2. Transfer Learning

Training deep learning models from scratch requires intensive computation and large amounts of labeled data. Today, with the development of computers with high speed and processing capacity, algorithms containing intensive calculations can be operated. Despite the powerful computers developed, training deep models from scratch takes a lot of time and is quite laborious. In addition, training the deep model with a dataset containing a small number of samples leads to an undesirable problem called overfitting. In the field of medical imaging, there is a limited amount of labeled data, so overfitting problem is highly likely. To overcome the above-mentioned problems, transfer learning approach has been proposed in the literature. Transfer learning is the transfer of a model that has been trained on a large dataset to solve a similar problem, together with its weights. Therefore, this approach eliminates the need to train the deep model from scratch and the large dataset required for training [54,55,56].

In this study, transfer learning approach was adopted because our pes planus dataset was not large enough. For this purpose, 8 pre-trained CNN models, namely MobileNetV2, AlexNet, DenseNet201, GoogleNet, VGG16, ResNet50, DarkNet19, and SqueezeNet, were used as feature extractor directly without fine tuning. These pre-trained CNN models were chosen for their success in medical image classification tasks [57]. The pseudocode of the feature generation process is presented in Algorithm 1. The extracted features are classified with conventional machine learning methods. In line with the results obtained, the best performing deep model (MobileNetV2) and classifier (SVM with cubic kernel) were selected for further classification.
**Algorithm 1**. The pseudocode of feature generation process.**Input:** Original X-ray image and 20 patches of original image. **Output:** 21,000-length feature vector extracted from popular pre-trained CNNs. ***for i = 1 to 21*** *alex_feats_ (1 + (1000 × (i − 1)):(i × 1000)) = alexnet(Xray_Image^i^)* *mobile_feats_ (1 + (1000 × (i − 1)):(i × 1000)) = mobilenetv2(Xray_Image^i^)* *dense_feats_ (1 + (1000 × (i − 1)):(i × 1000)) = dense201(Xray_Image^i^)* *google_feats_ (1 + (1000 × (i − 1)):(i × 1000)) = googlenet(Xray_Image^i^)* *vgg_feats_ (1 + (1000 × (i − 1)):(i × 1000)) = vgg16(Xray_Image^i^)* *resnet_feats_ (1 + (1000 × (i − 1)):(i × 1000)) = resnet50(Xray_Image^i^)* *darknet_feats_ (1 + (1000 × (i − 1)):(i × 1000)) = darknet19(Xray_Image^i^)* *squeeze_feats_ (1 + (1000 × (i − 1)):(i × 1000)) = squeezenet(Xray_Image^i^)* ***end for***

### 3.4. Iterative Feature Selection and Classification

Feature selection is defined as the process of selecting the most meaning or dominant features from the input predictor variables. Feature selection process reduces computational load, training time, and overfitting. Moreover, it may improve classification accuracy. In this study, the most important features were selected from the generated features by using the proposed iterative ReliefF algorithm [58], a filter-based feature selection algorithm. ReliefF is an improvement of the original Relief [59] algorithm developed by Kira and Rendell in 1992. The original Relief algorithm cannot handle incomplete and noisy data. Moreover, this method is limited to solving two-class problems only. The ReliefF algorithm, on the other hand, can deal with noisy or incomplete data and multi-class problems. The ReliefF algorithm first selects a random instance of $R_{i}$. After that, it searches its k nearest hits $H_{j}$ from the same class and its k nearest misses $M_{j}\left(C\right)$ from each of the different classes. Finally, it updates $W\left[A_{i}\right]$ for all features  A relying on their values for $R_{i}$, hits $H_{j}$ and misses $M_{j}\left(C\right)$. The update process is mathematically given as Equation (1).

(1) $$W\left[A_{i}\right]=W\left[A_{i}\right]-\overset{k}{\underset{j=1}{\sum }}diff\left(A_{i},R_{i},H_{j}\right)/m\;\left(\mathrm{dot}\right)\;k+\underset{C\neq Class\left(R_{i}\right)}{\sum }\left[\frac{P\left(C\right)}{1-P\left(class\left(R_{i}\right)\right)}\;\overset{k}{\underset{j=1}{\sum }}diff\left(A_{i},R_{i},M_{j}\left(C\right)\right)\right]/m\;\left(\mathrm{dot}\right)\;k\;\;\;$$

where $W\left[A_{i}\right]$ represents the weights of $i_{th}$ feature, m is the process cycle, $P\left(.\right)$ is the prior probability of C and diff defines the distance for discrete features as Equation (2).

(2) $$diff\left(A,I_{1},I_{2}\right)=\left\{\begin{array}{c} 0,\;if\;value\;\left(A,I_{1}\right)=value\;\left(A,I_{2}\right)\\ 1,\;if\;otherwise \end{array}\right.$$

where $I_{1}=R_{i}$, $\;I_{2}$ is either H or  M.

In this study, contrary to the literature, iterative ReliefF algorithm is proposed instead of ReliefF algorithm in feature selection process. The pseudocode of the proposed iterative ReliefF feature selection algorithm is presented in Algorithm 2. Accordingly, firstly, the features extracted from MobileNetV2, which is the best deep feature extractor, are normalized since the ReliefF algorithm is a neighborhood-based method. For normalization, the minimum–maximum normalization technique was used. Then, the normalized features and class labels were fed into the ReliefF. Thus, the indices and weights of the features were calculated for the 10 nearest neighbors. Afterwards, the features ranked in order of importance were iteratively evaluated with the best performing classifier, that is, using the SVM with cubic kernel (cubic SVM). The hyperparameters of SVM training are presented in Table 2. Finally, the minimum loss value and indices were calculated according to the evaluation results, and the features with the minimum loss value were selected. In the training and testing strategy of SVM is selected as 10-fold cross-validation. At the end of the feature selection process, the feature vector size was reduced from 1400 × 21,000 to 1400 × 1312.
**Algorithm 2**. The pseudocode of feature selection process.**Input:** Extracted features from MobilenetV2 (*mobilefeats*) and actual output(*actual_output*) **Output:** Classification accuracy(*accuracy*) *Feats = mobilefeats;* *Y = actual_output;* *normalized_mobilefeats_ = (feats − min(feats))./(max(feats) − min(feats));* *[idx, weights] = ReliefF(normalized_mobilefeats_,Y,10);* ***for***
***i = 1:21,000*** *selected_feats_ = feats(:,idx(1:i));* *loss(i) = QSVM(selected_feats_,Y,10);* ***end for*** *[idx_minloss_,lossvalue] = min(loss);* *accuracy= 1-QSVM(feats(:,(1:idx_minloss_)));*

## 4. Experimental Results and Discussion

In this section, experimental studies for automated and high accuracy diagnosis of pes planus and the results obtained from the proposed model are given. All coding was performed on a laptop equipped with 16 GB RAM, NVIDIA GeForce RTX 3050 GPU and Intel(R) Core(TM) i7-11800H CPU and MATLAB 2022a software. Initially, in the preprocessing stage, data augmentation was performed using geometric transformation-based methods to avoid overfitting and to increase the generalization ability of the model to be developed. Thus, the number of images in the collected dataset was augmented from 842 to 1400 and the number of images in each class was balanced to be equal. Since our dataset after data augmentation was not large enough, the transfer learning strategy was used in this study. For this purpose, the original X-ray images were first fed into 8 popular pre-trained CNN models, namely MobileNetV2, AlexNet, DenseNet201, GoogleNet, VGG16, ResNet50, DarkNet19 and SqueezeNet. Thus, 1400 × 1000 features were extracted for the entire dataset, with 1000 features for each original image in our dataset. Afterwards, the extracted deep features were evaluated by 7 conventional machine learning classifiers using 10-fold cross validation. The classification accuracy metric was used in the performance evaluation and the obtained accuracy scores are presented in Table 3. While the rows of Table 3 represent the 1000 features extracted from the pre-trained CNN models, the columns represent the machine learning classifiers in which these features are classified. Accordingly, the best performance was obtained from the SVM with cubic kernel for 1000 features extracted from MobileNetV2. The MobileNetV2-cubic SVM-based this model achieved 89.6% classification accuracy.

**Figure 5:**
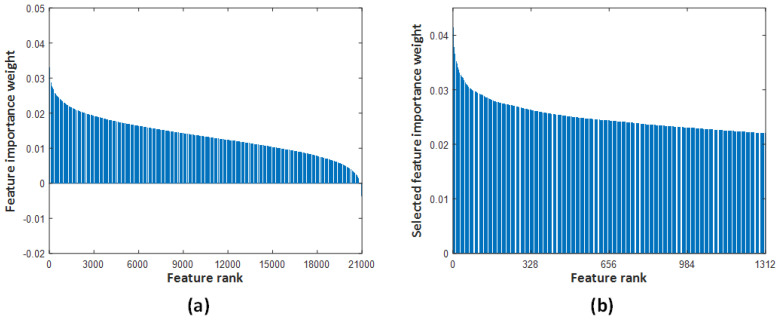
The feature selection process. (**a**) Weight value calculated by ReliefF algorithm for 21,000 features using 10 nearest neighbors. (**b**) The weight of the 1312 most important features selected from 21,000 features by the proposed iterative ReliefF algorithm.

**Figure 6:**
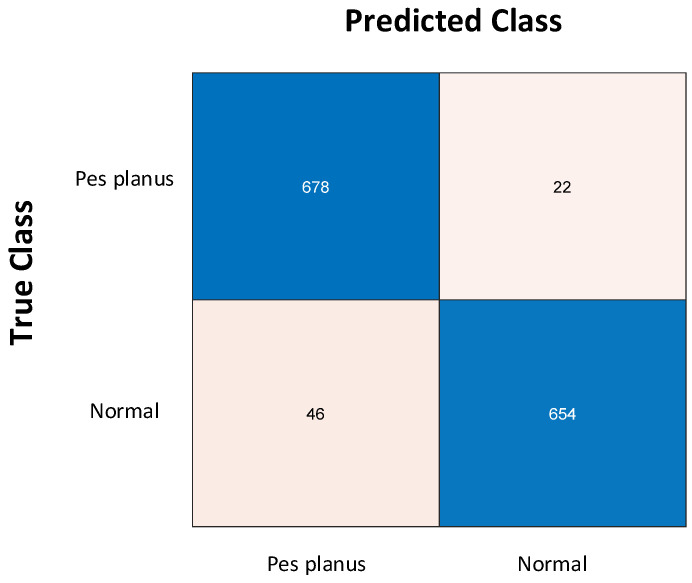
Confusion matrix of the proposed pyramidal-MobileNetV2-cubic SVM-based model.

In this study, it is aimed to develop a high-accuracy model to classify X-ray images normal or pes planus. For this purpose, the effectiveness of the pyramidal patch-based image division technique, which is commonly used in vision transformers and multilayer perceptron mixers (MLP-mixers) [60], was investigated. First, the original X-ray images were resized to 512 × 512. The resized images were then divided into 4 and 16 patches of 256 × 256 × 3 and 128 × 128 × 3 sizes, respectively, without overlapping. Thus, a total of 21 images were obtained for each image, including 20 patches and one original X-ray image. Then, the 21 images obtained for each image were fed to the best performing pre-trained MobileNetV2, and 21,000 features were extracted from the Logits layer. Thus, 1400 × 21,000 features were extracted for the entire dataset. Since the size of features extracted from MobileNetV2 is very large, feature selection was performed to reduce the computational load of the model to be developed and to increase the classification accuracy. For this purpose, the 1312 most important features were selected by running the proposed iterative ReliefF algorithm for 10 nearest neighbors. Figure 5a shows the weight of features that the original ReliefF algorithm has calculated for 21,000 features using 10 nearest neighbors. Figure 5b shows the weight of the most important 1312 features selected among 21,000 features by the proposed iterative ReliefF algorithm. As a result of the feature selection process, the size of the features has been reduced from 1400 × 21,000 to 1400 × 1312. Finally, the most important features selected are classified with cubic SVM. Accordingly, the pyramidal MobileNetV2-cubic SVM-based model achieved 95.14% accuracy using 10-fold cross-validation. The classification accuracy is calculated with Equation (3) over the confusion matrix shown in the Figure 6. In addition, precision, recall, and F1-score metrics is calculated to evaluate the proposed model using Equations (4)–(6), respectively. The proposed model yielded 96.86% recall, 93.65% precision, and 95.22% F1-score.

(3) $$accuracy=\frac{TP+FP}{\left(TP+TN+FP+FN\right)}$$

(4) $$precision=\frac{TP}{\left(TP+FP\right)}$$

(5) $$recall=\frac{TP}{\left(TP+FN\right)}$$

(6) $$F1-score=\frac{2*\left(precision*recall\right)}{\left(precision+recall\right)}$$

Here, true-positive (*TP*) is the number of correctly classified normal images and true-negative (*TN*) is the number of correctly classified pes planus images. False-positive (*FP*) and false-negative (*FN*) are the misclassified pes planus and normal images, respectively.

In addition, the performance of the pyramidal feature extraction and the proposed iterative ReliefF algorithm in other popular pre-trained CNN models were investigated. For this purpose, 21 images (original X-ray image-20 patches) obtained in a pyramidal fashion were fed to 7 pre-trained CNN models, namely AlexNet, DenseNet201, GoogleNet, VGG16, ResNet50, DarkNet19, SqueezeNet, and 21,000 features were extracted for each 21 images. Thus, 1400 × 21,000 features were extracted for the entire dataset from each pre-trained deep model. Then, the most important features were selected among the features extracted by running the iterative ReliefF algorithm and classified with cubic-SVM using 10-fold cross validation. To compare the proposed pyramidal-MobileNetV2-cubic SVM-based model with other models, accuracy, recall, precision, and F1-score metrics were calculated and the comparative results are presented in Table 4.

According to Table 3 and Table 4, pyramidal feature extraction from pre-trained CNN models, followed by running the iterative ReliefF algorithm, improved the classification accuracy by 5.54% for MobileNetV2, 8.04% for AlexNet, 5.90% for DenseNet201, 5.68% for GoogleNet, 7.71% for VGG16, 6.39% for ResNet50, 6.17% for DarkNet19, and 5.32% for SqueezeNet, respectively. In addition, the proposed pyramidal-MobileNetV2-cubic SVM-based model again showed the best performance compared to other models. Moreover, the fact that the MobileNet architecture is a lightweight CNN model compared to other CNN architectures minimizes the computational load of the proposed model and the need for powerful hardware. The high classification accuracy obtained from the proposed model indicates that the model can be used as an auxiliary tool for diagnosing pes planus in the clinic. To the best of our knowledge, model, method, or any CAD system has not been reported so far for the automatic and artificial intelligence-based diagnosis of pes planus from X-ray image. Therefore, in this study, a new dataset consisting of X-ray images was collected and a novel deep model based on pyramidal-MobileNetV2-cubic SVM was proposed. The proposed model could not be discussed with the literature, since no studies related to the subject were found in the literature.

The major limitation of this study is that the images in the dataset used in the study were collected from a single hospital and device. This may limit the generalization ability of the proposed model. In the future study, it is planned to expand the dataset by collecting X-ray images from different devices and hospitals. In addition, since the collected dataset was not large enough, a deep model could not be developed from scratch for automated pes planus diagnosis. Therefore, the transfer learning strategy was used in this study. However, thanks to the large dataset that is planned to be collected in the future, a special deep model for automated pes planus diagnosis will be developed and trained from scratch.

### Comparison with MLP-Mixers

This subsection involves a comparison of the performance between our proposed pyramidal-MobileNetV2-cubic SVM-based model and two variants of the MLP-Mixer models (S/32 and L/32), which have shown potential in various image classification tasks [60]. All experiments and coding related to MLP-Mixer were conducted in Python using the Timm library [61]. Our proposed model is similar to MLP Mixers in terms of dividing images into patches, which is why we conducted this comparison. Since our dataset is not large enough, we utilized pre-trained MLP-Mixer variants trained on the ImageNet dataset. This allows us to leverage the learned features from the pre-trained models while adapting them to our problem. To adapt the MLP-Mixer models for our specific task, we made modifications to the pre-trained MLP-Mixer models. Specifically, we replaced the original fully connected layer with a new layer that includes dropout at a rate of 0.3 and a linear layer with two output units, which corresponds to the two classes in our classification problem (normal or pes planus). To fairly compare the performance, we employed the same dataset for both models, splitting it into 70% for training, 15% for validation, and 15% for testing. Afterward, we fine-tuned the MLP-Mixer models for 250 epochs, using two different sets of hyperparameters, as described in Table 5. 

To evaluate and compare the models’ performance, we used metrics such as classification accuracy, recall, precision, and F1-score. Table 6 presents a comprehensive comparison of the results obtained from the MLP-Mixer models using both sets of hyperparameters and the performance of our proposed pyramidal-MobileNetV2-cubic SVM-based model. 

According to the results presented in Table 6, our proposed pyramidal-MobileNetV2-cubic SVM-based model outperforms in comparison to the MLP-Mixer models. Among the MLP variants, S/32 fine-tuned with set1 of hyperparameters achieved the best result with a test accuracy of 88.63%. In addition to achieving better performance than MLP models, our proposed model is computationally simpler and does not require fine-tuning. These indicates that our proposed model is more suitable for the task of classifying X-ray images as normal or pes planus, outperforming the MLP-Mixer approaches.

## 5. Conclusions

In recent years, many artificial intelligence-based systems have been developed as a result of rapid developments in artificial intelligence and especially deep learning. These systems produce smart solutions to complex problems. As the flagship of deep learning, CNNs have shown tremendous success in image classification and segmentation tasks. Due to this success of CNNs, many CAD systems have been developed and continue to be developed to assist the physician, reduce the workload, eliminate misinterpretation and provide an effective and accurate treatment. In addition, the development of a lightweight, high-accuracy, and reliable model is still one of the hot topics of research. Many artificial intelligence-based models have been proposed for the radiological image classification task in the studies. However, to the best of our knowledge, deep learning techniques have not been applied so far to diagnose pes planus from X-ray images. Therefore, in this study, a novel, automated, high-accuracy, reliable pyramidal-MobileNetV2-cubic SVM-based deep model using X-ray image, which is the standard imaging method for pesplanus diagnosis, is proposed. For this purpose, weight-bearing X-ray images of 439 patients were collected and the images were labeled as normal and pes planus by the specialist radiologists. The proposed pyramidal-MobileNetV2-cubic SVM-based model achieved 95.14% accuracy. The high classification accuracy obtained indicates that the proposed model can be used as an auxiliary tool in the diagnosis of pes planus. Moreover, this system can assist the expert in the decision-making process and reduce the workload. In future studies, it is aimed to develop a model with higher accuracy. In addition, it is considered to expand our dataset by collecting weight-bearing X-ray images from different hospitals.

## Figures and Tables

**Table 1:** Demographics of participants and dataset characteristics.

Participant Features	Category	Pes Planus		Normal	
		Patient, n	Image, n	Patient, n	Image, n
Age	14–24	94	188	98	196
	25–35	78	156	88	176
	35–47	29	58	34	68
Gender	Male	171	342	180	360
	Female	30	60	40	80

**Table 2:** The hyperparameters used for SVM training.

Hyperparameters	Value
Kernel function	Polynomial
Polynomial Order	3
Kernel Scale	auto
Box Constraint	1
Standardize	True

**Table 3:** Accuracy scores (%) of features extracted from popular CNN models with conventional machine learning classifiers.

Pre-Trained CNN Models	LD	QSVM	CSVM	Fine KNN	BoT	BaT	MNN
AlexNet	86.6	85.4	85.6	77.9	81.3	81.3	83.9
Densenet201	86.6	88.2	88.1	83.0	82.7	80.9	87.4
GoogleNet	88.3	88.0	88.4	82.0	82.3	82.3	84.0
VGG16	85.9	84.6	84.0	81.6	79.8	78.5	82.8
MobileNetV2	82.4	88.7	89.6	83.3	82.1	83.4	86.8
Resnet50	85.9	88.7	87.9	82.8	83.2	81.1	86.5
DarkNet19	85.4	87.5	86.4	79.5	83.5	83.0	85.3
SqueezeNet	88.0	86.6	85.6	80.1	80.4	79.4	85.4

LD: linear discriminant; QSVM: SVM with quadratic kernel; CSVM: SVM with cubic kernel; KNN: k-nearest neighbors; MNN: medium neural network; BoT: boosted trees; BaT: bagged trees.

**Table 4:** Performance comparison of proposed pyramidal MobileNetV2-cubic SVM-based model and other pyramidal-pre-trained CNN model-cubic SVM-based models.

Metod	Accuracy (%)	Recall (%)	Precision (%)	F1-Score (%)
**Proposed Pyr. MobilenetV2**	95.14	96.86	93.65	95.22
Pyr. AlexNet	93.64	94.14	93.21	93.67
Pyr. DenseNet201	94.00	94.43	93.63	94.03
Pyr. GoogleNet	94.08	95.14	93.15	94.13
Pyr. VGG16	91.71	91.00	92.32	91.65
Pyr. ResNet50	94.29	95.14	93.54	94.33
Pyr. DarkNet19	92.57	91.86	93.19	92.52
Pyr. SqueezeNet	90.93	91.57	90.41	90.99

**Table 5:** Hyperparameters for the MLP-Mixer models.

Hyperparameters	Set 1	Set 2
Optimizer	SGD	Adam
Learning Rate	0.01	0.01
Momentum	0.9	-
Weight Decay	1 × 10^−4^	1 × 10^−4^
Scheduler	CosineAnnealingWarmRestarts	ReduceLROnPlateau
Scheduler Parameters	T_0_: 250, T_mult_: 1, eta_min: 0.0001	Patience: 20, Factor: 0.1
Epoch	250	250
L1 Regularization	1 × 10^−5^	1 × 10^−5^
Gradient Clipping	Max norm:1	Max norm:1

**Table 6:** Performance comparison of the proposed pyramidal-MobileNetV2-cubic SVM-based model and MLP-Mixer models.

Metod	Accuracy (%)	Recall (%)	Precision (%)	F1-Score (%)
**Proposed Pyr. MobilenetV2**	95.14	96.86	93.65	95.22
MLP-Mixer S/32 (Set 1)	88.63	89.81	88.18	88.99
MLP-Mixer B/32 (Set 1)	83.89	88.18	82.2	85.09
MLP-Mixer S/32 (Set 2)	81.99	86.67	79.13	82.73
MLP-Mixer B/32 (Set 2)	88.15	95.74	81.08	87.80

## Data Availability

The datasets collected during and/or analyzed during the current study are available from the corresponding author on reasonable request.
